# Alleviation by Mahuang Fuzi and Shenzhuo Decoction in High Glucose-Induced Podocyte Injury by Inhibiting the Activation of Wnt/*β*-Catenin Signaling Pathway, Resulting in Activation of Podocyte Autophagy

**DOI:** 10.1155/2020/7809427

**Published:** 2020-09-03

**Authors:** Haoran Dai, Fei Liu, Xinping Qiu, Wenbin Liu, Zhaocheng Dong, Yingmin Jia, Zhendong Feng, Zhiyuan Liu, Qihan Zhao, Yu Gao, Zihan Zhang, Chang Gao, Songge Sun, Xuefei Tian, Baoli Liu

**Affiliations:** ^1^Department of Nephrology, Shunyi Hospital, Beijing Traditional Chinese Medicine Hospital, Station East 5, Shunyi District, Beijing 101300, China; ^2^Beijing University of Chinese Medicine, No. 11, North Third Ring Road, Chaoyang District, Beijing 100029, China; ^3^Beijing Hospital of Traditional Chinese Medicine Affiliated to Capital Medical University, 23 Meishuguanhou Street, Dongcheng District, Beijing 100010, China; ^4^Capital Medical University, No. 10, Xitoutiao, You'anmenwai, Fengtai District, Beijing 100069, China; ^5^Beijing Chinese Medicine Hospital Pinggu Hospital, No. 6, Pingxiang Road, Pinggu Town, Pinggu District, Beijing 101200, China; ^6^Shandong First Medical University, No. 619, Changcheng Road, Tai'an, Shandong 271000, China; ^7^Section of Nephrology, Department of Internal Medicine, Yale School of Medicine, New Haven, Connecticut 06510, USA

## Abstract

**Background:**

Organ fibrosis is a common endpoint of a variety of diseases. Many studies have shown that the pathogenesis of diabetic kidney disease (DKD) is related to the excessive activation of the Wnt/*β*-catenin signaling pathway on podocytes, so the treatment of DKD starts from this signaling pathway. At the same time, DKD, as a metabolic disease, has many connections related to podocyte autophagy.

**Objectives:**

We experimented the effects of Mahuang Fuzi and Shenzhuo decoction (MFSD) which is the combination of Mahuang Fuzi decoction and Shenzhuo decoction in traditional Chinese medicine compounds used “The Golden Chamber” in high glucose-induced podocytes, determined whether this effect was related to Wnt/*β*-catenin signaling pathway, and further investigated the relationship between this effect and autophagy.

**Methods:**

The mice podocytes were stimulated by using 30 mmol/L of high glucose and serum containing MFSD or Wnt/*β*-catenin signaling pathway inhibitor DKK1 (100 ng/ml) was used to intervene podocytes before high glucose stimulation. Podocyte injury-related proteins, Wnt/*β*-catenin signaling pathway-related proteins, and autophagy-related proteins were detected by using western blotting and immunofluorescence analysis.

**Results:**

Our results showed that DKK1 and MFSD treatment significantly upregulated the protein expressions of nephrin, podocin, podocalyxin, and podoplanin in high glucose-induced podocytes and downregulated the *β*-catenin protein expression. Furthermore, the protein expressions of beclin1, LC3B, and P62 were also significantly increased in high glucose-induced podocytes.

**Conclusion:**

Our experiments confirmed that the destruction of podocytes in DKD is related to the excessive activation of Wnt/*β*-catenin signaling pathway and the inhibition of autophagy after activation. MFSD treatment can inhibit the activation of Wnt/*β*-catenin signaling pathway in podocytes stimulated by high glucose and helpful in reducing the podocyte injury. This protective mechanism can be related to the enhancement of podocyte autophagy by MFSD treatment.

## 1. Introduction

Diabetic kidney disease (DKD) is one of the major complications of diabetes [1]. The traditional therapies of DKD are mostly based on hypoglycemic, antihypertensive, diet, and lifestyle [2]. However, the efficacy is not fully satisfactory [3], and further research should be carried out. The occurrence of DKD is related to destruction of podocyte and alteration in the structure of the podocyte under high glucose levels, including podocyte hypertrophy, podocyte epithelial-mesenchymal transdifferentiation, podocyte detachment, and podocyte apoptosis [4, 5], which leads to the destruction of glomerular filtration barrier and results in proteinuria [6, 7]. Therefore, this research aims to explore a new approach to alleviate podocyte injury which is an important therapeutic strategy [8].

Wnt/*β*-catenin signaling pathway is a common signaling pathway regulating cell proliferation, *β*-catenin is the main effector protein [9, 10], and it is stably expressed low in highly differentiated kidney cells of mammals [11, 12]. When the cells are stimulated, the Wnt/*β*-catenin signaling pathway is aberrantly activated [13]. Abnormal activation of the Wnt/*β*-catenin signaling pathway further amplifies destruction when podocytes are damaged. Many studies have reported abnormal activation of the Wnt/*β*-catenin signaling pathway in podocytes [14–16]. Autophagy is a highly conserved protective mechanism present in eukaryotic cells which is involved in the maintenance of cell turnover and cell homeostasis by degradation of lysosomal proteins, clearance of damaged structures, or overexpressed proteins [17–19]. Extensive studies have shown that autophagy was regulated by a variety of mechanisms, including activation of the Wnt/*β*-catenin signaling pathway [20, 21].

Cumulative studies have shown that a variety of traditional Chinese medicines (TCMs) are used to treat various diseases including chronic kidney diseases (CKD) [22–24]. A compelling evidence has been demonstrated that many crude extracts and combination of different natural products can inhibit the Wnt/*β*-catenin pathway in CKD [25, 26]. Mahuang Fuzi decoction and Shenzhuo decoction, two unique traditional Chinese medicine mixtures, were used to treat tissue fibrosis-associated disease clinically, for more than 1800 years ago. Organ fibrosis is a common endpoint of diverse diseases, and TCM was widely used for antifibrosis [27, 28]. Mahuang Fuzi and Shenzhuo decoction (MFSD) is used to treat CKD and improves renal fibrosis such as membranous nephropathy and has achieved remarkable efficacy; furthermore, it was also applied in the treatment of DKD. We had done research that whether MFSD can alleviate the destruction of podocytes due to high glucose levels, which can be related to Wnt/*β*-catenin and autophagy pathway.

## 2. Materials and Methods

### 2.1. Animals

40 male Sprague Dawley (SD) mice, aged 6–8 weeks old, weighing between 200 and 220 g, were purchased from Beijing Huakang Biotechnology Co., Ltd. The number of mice was determined according to the experimental needs. Standard rodent chow and water were obtained freely by feeding all the mice in 12-hour/12-hour light/dark conditions without any other specific pathogens. At the time of the experiment, mice were anesthetized according to the body weight of the mice by intraperitoneal injection of 1% sodium pentobarbital at 1 ml/100 g. The blood of the mice was obtained through the abdominal artery under general anesthesia, and finally, the mice died due to excessive blood loss. All of the experimental procedures involving animals were approved by the Experimental Animal Welfare Ethics Committee of the Beijing Institute of Chinese Medicine, Ethics No. 2018090102.

### 2.2. Preparation of MFSD-Serum (MFSDS)

The specific information such as the composition and dosage of MFSD is shown in Table 1. The mice were perfused with nonfried granules, and the dosage in mice was adjusted according to the conversion coefficient of human adult's applied medication. All nonfried granules were purchased from Guangdong Party Pharmaceutical Co., Ltd.

20 SD mice were perfused with MFSD for one week to obtain serum containing MFSD (MFSDS), and another 20 SD mice were orally administered with normal saline for one week to obtain control serum (CS). A week later, mice blood was obtained and centrifuged at 3000 rpm for 10 min at 4°C, and the serum was inactivated at 56°C for 30 min, filtered with a 0.22 *μ*m filter, and stored at −20°C.

### 2.3. Cell Culture

Conditional immortal mouse glomerular podocytes (GPC) were presented by Professor Maria Pia Rastaldi of S. Carlo Hospital, University of Milan, Italy. The podocytes were cultured in RPMI 1640 (Gibco, USA) containing 10% fetal bovine serum and 50 u/ml interferon-*γ* at 33°C in an incubator with 5% CO_2_, The podocytes passaged until the cells were in good growth state and covered 70% to 80% of the culture dishes. In the interferon-containing 1640 medium, the culture was incubated at 37°C in 5% CO_2_, and the cells were fully differentiated after 10 to 14 days, which were used for the experiment. These cells were divided into four groups: (1) control group with 10% CS, (2) high glucose group with 10% CS, (3) DKK1 group with 10% CS, and (4) MFSD group with 10% MFSDS.

### 2.4. Immunofluorescence Analysis

The cultured and well-prepared podocytes were fixed with 4% paraformaldehyde for 15 minutes and washed three times with PBS, each time for 5 minutes. The membrane was diafiltered with 0.5% Triton-100 for 30 min at room temperature and washed three times with PBS for 5 minutes each time. 5% BSA was used to block cells at room temperature for 1 hour and then primary antibody was added and incubated overnight at 4°C. After that, the primary antibody was discarded and washed three times with PBS, each time for 5 minutes, and the cytoskeleton was stained with phalloidin (Thermo Fisher Scientific, USA). The target protein was stained with Alexa Fluor 594 (Thermo Fisher Scientific, USA), and the nucleus was stained with 5%DAPI (Thermo Fisher Scientific, USA). At last, the specimen was observed under a confocal microscope (LSM 800, ZEISS, Germany).

### 2.5. Western Blotting

Western blot experiments were performed according to the instructions of the WES kit (Protein Simple, USA). The cultured and intervened podocytes were collected in groups, and then, an appropriate amount of RIPA lysate (Thermo Fisher Scientific, USA) was added at 4°C, fully lysed for 30 minutes, and centrifuged at 14,000 rpm, for 5 minutes, and the supernatant was collected, and the BCA quantitative assay kit (Thermo Fisher Scientific, USA) was used to determine the protein concentration. 1.5 *μ*g of each well was loaded, and the antibody dilution, primary antibody, secondary antibody, chemiluminescence solution, and wash buffer were sequentially added to the sample plate, and experiment was performed on the equipment. The remaining part was completed automatically by the equipment after adjusting the relevant settings.

### 2.6. Statistical Analysis

According to the obtained data, a statistical analysis plan was made and SPSS Statistics 23.0 software was applied for analysis. Data that conformed to the normal distribution were expressed as mean ± standard deviation, and data that did not conform to the normal distribution were expressed as median (quartile spacing). One-way analysis of variance was done to calculate the difference between experimental groups in GraphPad Prism8 (GraphPad Software, San Diego, CA). *P* < 0.05 was considered statistically significant.

## 3. Results

### 3.1. Effects of MFSD-Serum on the Morphology of Podocytes

**Figure 1:**
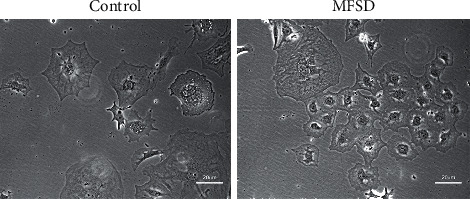
Effect of MFSD on the morphology of podocytes. The morphology of podocytes cultured by 10% MFSDS for 24 hours was observed under light microscope. The cells differentiate after being cultured at a 37°C incubator with 5% CO_2_ for 10 days to 14 days, the adherent growth area of the cells increases significantly, and a large number of secondary protrusions protrude from the main body of the cell body, making the cells appear “dendritic” or “flower-like.”

We observed the morphology of the MFSD group after a 24-hour intervention under light microscopy (Figure 1). The cells were differentiated after being cultured at 37°C in an incubator with 5% CO_2_ for 10 days to 14 days, the adherent growth area of the cells increases significantly, and a large number of secondary protrusions protrude from the main part of the cell body, making cells to appear “dendritic” or “flower-like.” There is no obvious difference in morphology between the two groups of podocytes. This shows that the MFSD-containing serum has no obvious effect on the morphology of podocytes.

### 3.2. DKK1 and MFSD Effectively Alleviates High Glucose-Induced Podocyte Injury

**Figure 2:**
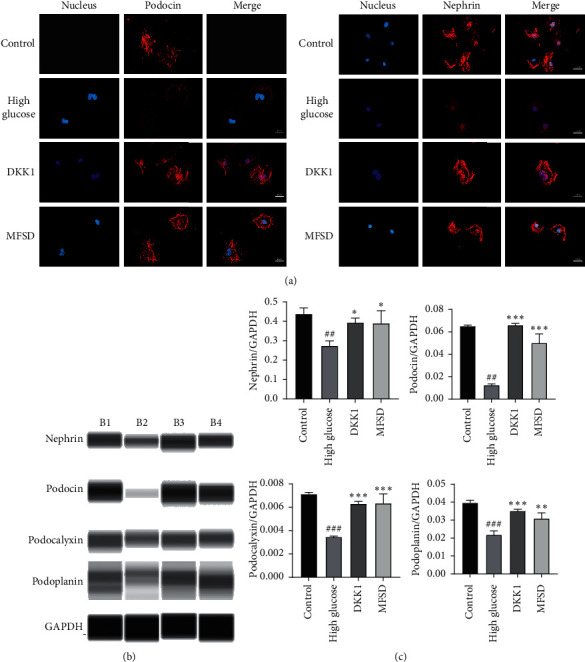
MFSD treatment significantly reduces the damage of high glucose on podocytes. (a) Immunofluorescences for podocin and nephrin of the control group, high glucose group, DKK1 group, and MFSD group; red represents the corresponding protein and blue represents the nucleus. (b) The protein expressions of podocin, nephrin, podocalyxin, and podoplanin were analyzed by western blot. B1 represents control group, B2 represents high glucose group, B3 represents DKK1 group, and B4 represents MFSD group. (c) Quantitative statistical analysis of western blot. The obtained value of podocin, nephrin, podocalyxin, and podoplanin was normalized to the GAPDH. ^#^*P* < 0.05, ^###^*P* < 0.01, and ^###^*P* < 0.001 compared with control group; ^*∗*^*P* < 0.05, ^*∗∗*^*P* < 0.01, and ^*∗∗∗*^*P* < 0.001 compared with high glucose group.

Before the intervention of high glucose (30 mmol/L) in podocytes, first of all, we cultured podocytes with DKK1 (100 ng/ml) or 10% MFSDS for 24 hours. Subsequently, podocytes were incubated with high glucose medium for 24 hours to cause destructive phenomena. After cell culture, the expression of podocyte-related proteins was analyzed by immunofluorescence and western blots. We found out that the expression levels of nephrin and podocin were significantly decreased in high glucose conditions. Western blots showed that the expression levels of nephrin, podocin, podocalyxin, and podoplanin in the DKK1 group and MFSD group also restored compared with the high glucose group (Figure 2). These results demonstrated that DKK1 and MFSD can effectively alleviate the destruction of podocyte caused by high concentration of glucose.

### 3.3. DKK1 and MFSD Treatment Inhibits Wnt/*β*-Catenin Signaling Pathway Activation

**Figure 3:**
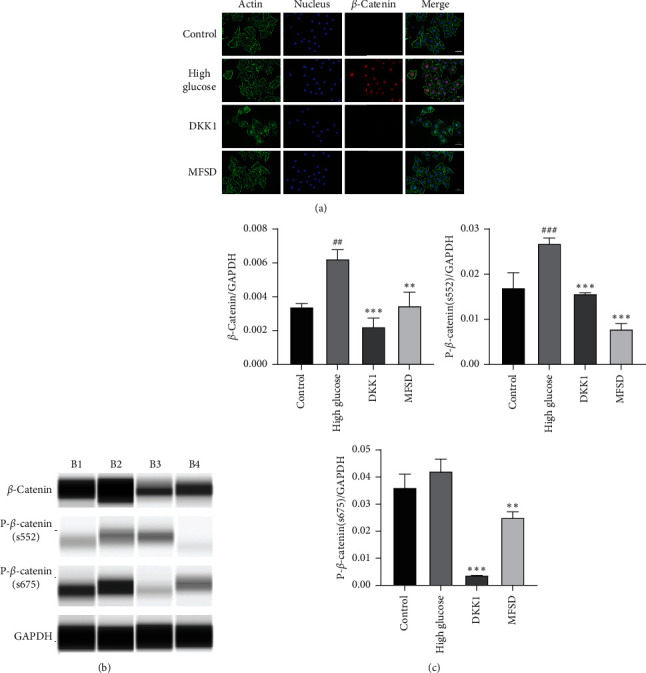
MFSD inhibits Wnt/*β*-catenin signaling pathway activation. (a) Immunofluorescence for the expression of *β*-catenin in the control group, high glucose group, and MFSD group; red represents *β*-catenin, green represents cytoskeleton, and blue represents nucleus. (b) The protein expressions of *β*-catenin, p-*β*-catenin (s552), and p-*β*-catenin (s675) were analyzed by western blot. B1 represents control group, B2 represents high glucose group, B3 represents DKK1 group, and B4 represents MFSD group. (c) Quantitative statistical analysis of western blot. The obtained values of *β*-catenin, p-*β*-catenin (s552), and p-*β*-catenin (s675) were normalized to the GAPDH.^#^*P* < 0.05, ^###^*P* < 0.01, and ^###^*P* < 0.001 compared with control group; ^*∗*^*P* < 0.05, ^*∗∗*^*P* < 0.01, and ^*∗∗∗*^*P* < 0.001 compared with high glucose group.

DKK1, as a Wnt/*β*-catenin signaling pathway inhibitor, showed an inhibitory effect on Wnt/*β*-catenin signaling pathway which was significant. In order to study the effect of MFSD on Wnt/*β*-catenin signaling pathway, we further observed the quantity of the key protein of *β*-catenin and phosphorylated *β*-catenin expression. The results showed that protein expression levels were significantly increased in the high glucose group, indicating abnormal activation of Wnt/*β*-catenin signaling pathway. Under the intervention of DKK1 or MFSD, their expressions were significantly inhibited, indicating that the activation of the Wnt/*β*-catenin signaling pathway was blocked (Figure 3).

### 3.4. DKK1 and MFSD Treatment Restores Podocyte Autophagy Induced by High Glucose

Our experiments confirmed that MFSD treatment can significantly inhibit the abnormal activation of Wnt/*β*-catenin signaling pathway and can reduce high glucose-induced podocyte injury. However, the mechanism remains enigmatic.

**Figure 4:**
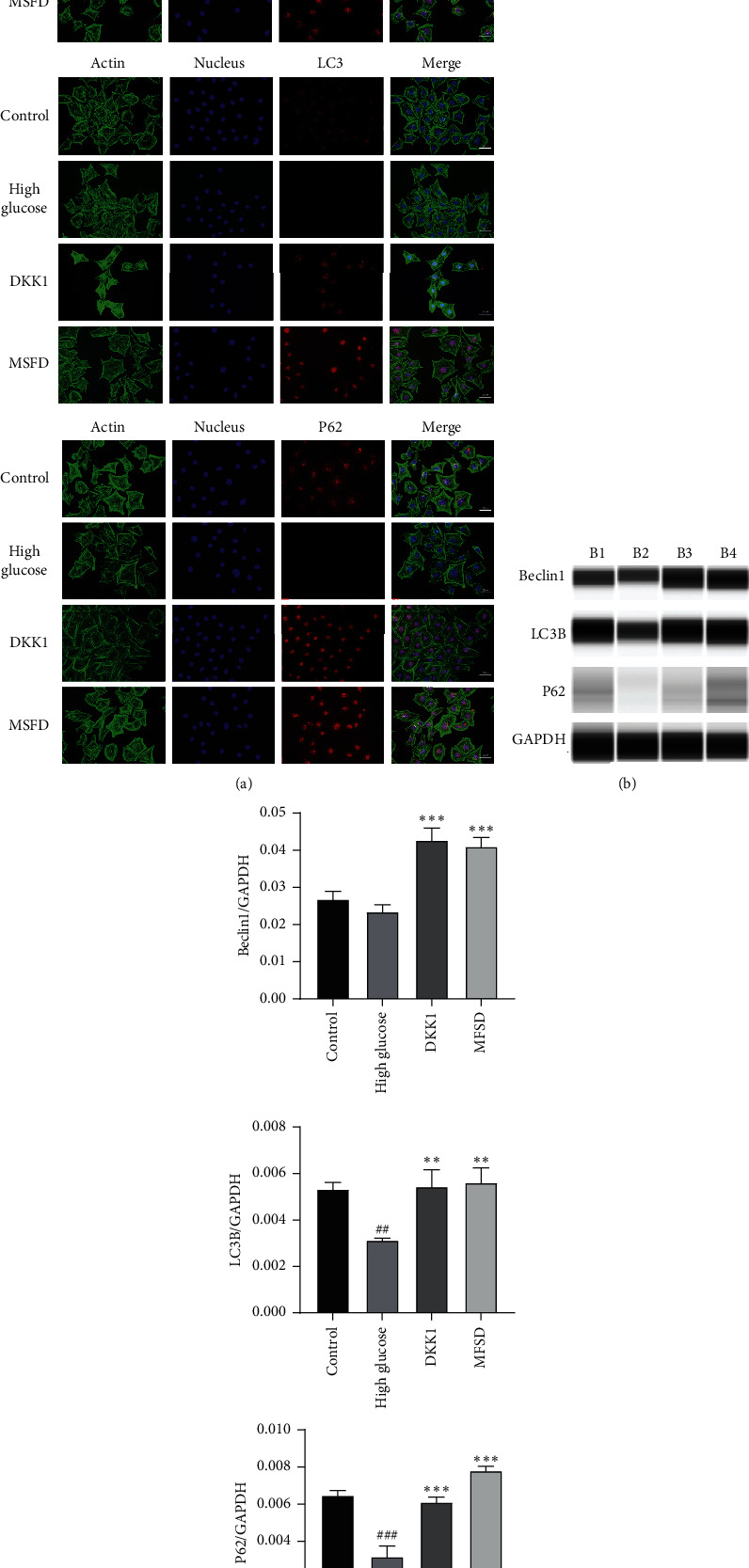
MFSD restores podocyte autophagy affected by high glucose. (a) Immunofluorescence for the expression of LC3B, beclin1, and p62/SQSTM1 in the control group, high glucose group, and MFSD group; red represents corresponding protein, green represents cytoskeleton, and blue represents nucleus. (b) The protein expressions of LC3B, beclin1, and p62/SQSTM1 were analyzed by western blot. B1 represents control group, B2 represents high glucose group, B3 represents DKK1 group, and B4 represents MFSD group. (c) Quantitative results of western blotting analysis. The obtained values of LC3B, beclin1, and p62/SQSTM1 were normalized to the GAPDH. ^#^*P* < 0.05, ^###^*P* < 0.01, and ^###^*P* < 0.001 compared with control group; ^*∗*^*P* < 0.05, ^*∗∗*^*P* < 0.01, and ^*∗∗∗*^*P* < 0.001 compared with high glucose group.

In order to explore whether DKK1 or MFSD treatment can regulate the autophagy in podocytes, we experimented the effect of DKK1 and MFSD on autophagy. To observe the status of autophagy after podocytes were intervened, we examined autophagy-related proteins LC3B, beclin1, and p62. These experimental results confirmed that the protein expressions of LC3B, beclin1, and p62 were significantly decreased in the high glucose group, indicating that the autophagy of podocytes was inhibited under high glucose conditions. Interestingly, the protein expressions of LC3B, beclin1, and p62 were significantly increased in the DKK1 and MFSD group, which represented an upregulation of podocyte autophagy levels. At the same time, there was no significant difference in the level of autophagy between the DKK1 group and the MFSD group (Figure 4).

## 4. Discussion

DKD is the most common cause of end-stage kidney disease worldwide. The pathogenic mechanisms are mostly understood [29–32]. However, current treatment methods such as hypoglycemic, dietary control, and life adjustment are not satisfactory [33]. We further need to develop a more effective therapy for DKD treatment. As traditional Chinese medicine treatment, many effective prescriptions and compounds from TCM have been used in the treatment of renal diseases [34–36]. Mahuang Fuzi decoction and Shenzhuo decoction are well-known prescriptions in TCM, and the important ingredients are Fu zi, Gan jiang, Ma huang, Fu ling, Bai zhu, and Gan cao. They are widely used in the clinical treatment of various diseases including renal diseases. Clinical findings confirmed that MFSD can effectively reduce edema, reduce proteinuria, and increase serum albumin in patients with DKD. But its molecular mechanism is still unknown; we firstly verify the protective effect of MFSD on podocytes *in vitro*.

Podocytes are unique, highly differentiated glomerular epithelial cells which are important components of the glomerular filtration barrier [37]. Further studies have shown that podocytes undergo a series of morphological and functional changes in diabetic nephropathy, mainly including hypertrophy, epithelial-mesenchymal transition differentiation, shedding, and apoptosis [5]. The experimental results showed that DKK1 and MFSD can effectively reduce high glucose-induced podocyte injury. Compared with the high glucose group, the protein expression levels of nephrin, podocin, podocalyxin, and podoplanin in the DKK1 and MFSD groups were significantly increased, which indicates that the injury of podocytes was inhibited. Hence, DKK1 and MFSD can effectively protect podocytes in conditions having high glucose levels.

The Wnt protein is a secreted glycoprotein containing a signal peptide and 23 or 24 conserved cysteine residues [38–40]. Wnt signaling pathway affects cell differentiation, proliferation, maturation, and viability [41–43]. Several studies have demonstrated the activation of Wnt/*β*-catenin signaling pathway in patients with CKD [44, 45]. In glomerular diseases such as DKD and azithromycin nephropathy, the Wnt family members of the glomerular podocytes are significantly upregulated and activate the downstream *β*-catenin, thereby activating the Wnt/*β*-catenin signaling pathway [46]. By inhibiting the Wnt/*β*-catenin signaling pathway, such as knocking out *β*-catenin in glomerular podocytes or using the Wnt/*β*-catenin signaling pathway to inhibit protein DKK1, it can significantly reduce podocyte injury, reduce proteinuria, and also protect the kidney [47–49], at the same time. Our experimental results also confirm the protective effect of inhibiting Wnt/*β*-catenin signaling pathway on podocytes.

Many small molecules from natural products have been demonstrated to target Wnt/*β*-catenin signaling pathway [25, 50, 51]. A number of small molecules from natural products can inhibit Wnt/*β*-catenin signaling pathway in podocytes [52, 53]. Since the mechanism of protective effect on podocytes by MFSD is not clear, we tentatively tested the activation of Wnt/*β*-catenin signaling pathway in podocytes. Interestingly, we found out that *β*-catenin and phosphorylated *β*-catenin were significantly increased in the high glucose group, indicating abnormal activation of the Wnt/*β*-catenin signaling pathway, which is consistent with previous studies [54]. The expression of these Wnt/*β*-catenin signaling pathway-related proteins was significantly reduced in the DKK1 and MFSD groups, which indicated that MFSD could inhibit the abnormal activation of Wnt/*β*-catenin signaling pathway. Combined with the previously available literature, we sort out that MFSD can reduce high glucose-induced podocyte injury by inhibiting the activation of Wnt/*β*-catenin signaling pathway.

However, it is not enough to explain that MFSD relies on excessive activation of the Wnt/*β*-catenin signaling pathway to alleviate high glucose-induced podocyte injury. Further studies are required to rule out the whole mechanism. In DKD, podocytes are in a high glucose environment for a long time. Under the stimulation of long-term high glucose, autophagy is inhibited, and accumulated reactive oxygen species, glycosylation end products, etc., cannot be eliminated in time, and podocyte apoptosis is finally induced. Our experimental results showed that the expression levels of autophagy-related proteins LC3B and beclin1 in the high glucose group were reduced, which means that autophagy in podocytes in the high glucose group was suppressed. Under normal circumstances, P62 participates in the process of autophagolysosome degradation of protein and P62 is also degraded by this process. When cell autophagy is impaired, P62 cannot be degraded smoothly and accumulates in the cell [55]. Strangely, our results showed that the expression of P62 in podocytes of the high glucose group was reduced. Therefore, further research is required to explain the reduction of P62 expression.

Studies have shown that activation of the Wnt/*β*-catenin signaling pathway plays a key role in autophagy [56, 57]. For example, the Wnt/*β*-catenin signaling pathway is involved in the regulation of autophagy in prostate cancer cells [58], preventing Wnt/*β*-catenin signaling pathway can promote the expression of autophagy in myocardium P19CL6 cells [59], while *β*-catenin inhibits autophagy by inhibiting the production of p62 [57]. Based on the reported results, we further tested the effect of DKK1 and MFSD on autophagy. It was noted that the protein expression levels of LC3B, beclin1, and p62 in the high glucose group were significantly decreased, which indicates that the level of autophagy was significantly decreased. At the same time, the intervention of DKK1 and MFSD upregulated the autophagy level in podocytes. This explains the reduction of P62 expression in podocytes of the high glucose group: when the Wnt/*β*-catenin signaling pathway is activated, *β*-catenin accumulates in the cytoplasm and eventually enters the nucleus affecting the transcription of P62, which results in insufficient amount of P62 in the cytoplasm during the process of autophagy.

**Figure 5:**
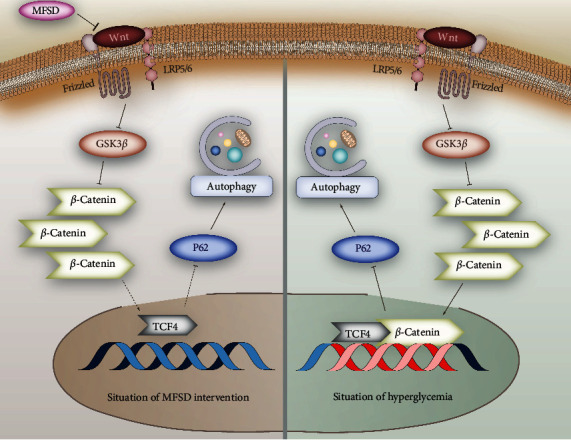
One of the possible mechanisms of MFSD to alleviate the damage of high glucose to podocytes. In high glucose environment, Wnt/*β*-catenin signaling pathway is activated, and *β*-catenin accumulates in cells and finally participates in nuclear transcription of downstream proteins, inhibits transcription and production of p62/SQSTM1, and restricts autophagy. MFSD inhibits the activation of Wnt/*β*-catenin signaling pathway and prevents the autophagy process from being restricted.

Regarding the above experimental results, we found out that the mechanism of high glucose damaging the podocytes involves signaling pathway and autophagy, and there is also a certain relation between signaling pathway and autophagy. We can also clearly see the protective effect of MFSD on podocytes induced by high glucose. This protective effect was likely to upregulate the autophagy of podocytes by inhibiting activation of Wnt/*β*-catenin signaling pathway. At the level of implementation, specifically, the protective mechanism can be such that long-term high glucose stimulation leads to abnormal activation of the Wnt/*β*-catenin signaling pathway, resulting in an increase in the expression of *β*-catenin, thereby inhibiting the production of p62, ultimately leading to inhibition of autophagy results in destruction of podocytes, and intervention by MFSD blocked the abnormal activation of the Wnt/*β*-catenin signaling pathway and upregulated the process of autophagy in podocytes (Figure 5).

## 5. Conclusion

The destruction of podocytes in DKD is related to the excessive activation of Wnt/*β*-catenin signaling pathway and the inhibition of autophagy after activation. Inhibition of Wnt/*β*-catenin signaling pathway can activate autophagy and can significantly reduce the destruction of podocytes in high glucose level. Our findings demonstrated that MFSD treatment can effectively alleviate the destruction of podocytes induced by high glucose and can also protect the podocytes by inhibiting the activation of the Wnt/*β*-catenin signaling pathway and simultaneously upregulating the autophagy level in podocytes, providing a new way of treating DKD.

## Figures and Tables

**Table 1:** The composition of MFSD.

	TCM name	Latin name	Part used	Serial number	Dosage (g/kg)
Ephedra	Ma huang	*Ephedra sinica Stapf*	Stem	Lot.7051972	0.36
Aconite	Fu zi	*Aconitum carmichaelii Debx.*	Lateral radix	Lot.6120142	0.42
Dried ginger	Gan jiang	*Zingiber oj-jicinale Rosc.*	Rhizome	Lot.7090862	0.9
Tuckahoe	Fu ling	*Poria cocos (Schw.) Wolf*	Sclerotium	Lot.7010742	0.27
Atractylodes	Bai zhu	*Atractylodes macrocephala Koidz.*	Radix	Lot.6126142	1.62
Licorice	Gan cao	*Glycyrrhiza uralensis Fisch*	Radix and rhizome	Lot.7021762	0.36

## Data Availability

The datasets used and/or analyzed during the current study are available from the corresponding author upon request.
